# Synergistic cytotoxicity of perifosine and ABT‐737 to colon cancer cells

**DOI:** 10.1111/jcmm.17636

**Published:** 2022-12-15

**Authors:** Barbora Adamová, Kamila Říhová, Jana Pokludová, Petr Beneš, Jan Šmarda, Jarmila Navrátilová

**Affiliations:** ^1^ Department of Experimental Biology, Faculty of Science Masaryk University Brno Czech Republic; ^2^ International Clinical Research Center St. Anne's University Hospital Brno Czech Republic

**Keywords:** ABT‐737, colon cancer, combined treatment, peeling analysis, perifosine, spheroids, synergism, tumour microenvironment

## Abstract

An acidic environment and hypoxia within the tumour are hallmarks of cancer that contribute to cell resistance to therapy. Deregulation of the PI3K/Akt pathway is common in colon cancer. Numerous Akt‐targeted therapies are being developed, the activity of Akt‐inhibitors is, however, strongly pH‐dependent. Combination therapy thus represents an opportunity to increase their efficacy. In this study, the cytotoxicity of the Akt inhibitor perifosine and the Bcl‐2/Bcl‐xL inhibitor ABT‐737 was tested in colon cancer HT‐29 and HCT‐116 cells cultured in monolayer or in the form of spheroids. The efficacy of single drugs and their combination was analysed in different tumour‐specific environments including acidosis and hypoxia using a series of viability assays. Changes in protein content and distribution were determined by immunoblotting and a “peeling analysis” of immunohistochemical signals. While the cytotoxicity of single agents was influenced by the tumour‐specific microenvironment, perifosine and ABT‐737 in combination synergistically induced apoptosis in cells cultured in both 2D and 3D independently on pH and oxygen level. Thus, the combined therapy of perifosine and ABT‐737 could be considered as a potential treatment strategy for colon cancer.

## 1 BACKGROUND

Cancer is one of the leading causes of premature death and the number of new cases continues to rise. In 2020, more than 19 million new cases were diagnosed, of which 10% are colorectal carcinomas (CRC).^1^ Although there are a number of genes whose mutations are typical of CRC (mainly *TP*53, *KRAS*, etc.),^2^ tumours are very heterogeneous and have many genetic and epigenetic alterations.^2^, ^3^

Approximately 15% of CRCs have abnormalities in the PI3K/Akt pathway, leading to overstimulation of cell growth and proliferation.^2^, ^4^, ^5^ Therapies targeting Akt are currently being developed and tested in clinical trials to inhibit this important survival‐promoting pathway.^6^ Perifosine is a synthetic alkyl phospholipid that inhibits Akt phosphorylation and mediates caspase‐activated cell death.^7^, ^8^ However, the activity of Akt inhibitors is highly pH‐dependent, as they lose efficacy under acidic conditions.^9^, ^10^ Unfortunately, an acidic environment and low tumour oxygen levels are hallmarks of cancer and contribute to cell resistance.^11^

Cell death is regulated by B‐cell lymphoma 2 (Bcl‐2) family proteins that share the Bcl‐2 homology domain (BH), which includes potent pro‐ (BH3) and anti‐apoptotic regulators.^12^ In healthy cells, BH3 are largely inactive and are blocked by binding with anti‐apoptotic members (Bcl‐2, Bcl‐xL). Upon an apoptotic stimulus, the BH3s are released by the anti‐apoptotic proteins and the apoptotic cascade is activated, leading to the release of cytochrome‐c from mitochondria.^13^, ^14^, ^15^ The anti‐apoptotic proteins are often upregulated in acidosis by altered PI3K/Akt signalling pathway.^16^ To overcome interfering features of the tumour environment and improve the efficacy and specificity of therapeutic strategies, multiple deregulated signalling pathways can be targeted.^17^, ^18^

ABT‐737, a BH3‐mimetic protein, was developed as an inhibitor of the anti‐apoptotic members of Bcl‐2. ABT‐737 effectively kills acute myeloid leukaemia, lung cancer, and glioblastoma cells.^19^, ^20^, ^21^ In addition, ABT‐737 showed a stronger effect against carcinoma cells under hypoxic and acidic conditions.^22^, ^23^, ^24^ However, both perifosine and ABT‐737 have been shown to be more effective in combination with other drugs than as a single treatment.^13^, ^25^, ^26^, ^27^, ^28^, ^29^ Therefore, in this study, we tested the simultaneous inhibition of the PI3K/Akt pathway and the Bcl‐2 protein family by combined treatment with perifosine and ABT‐737 in colon cells, focusing on their cytotoxicity under acidic and hypoxic conditions. We investigated the efficacy of these drugs both with cells cultured in a monolayer and in the form of spheroids, as 3D tumour models allow a better simulation of the real tumour‐specific environment.^30^, ^31^

## 2 METHODS

### 2.1 Cell cultures and chemicals

HT‐29 and HCT‐116 human adenocarcinoma cells were obtained from LGC Standards (Teddington, UK). Cells were grown in humidified incubator set to 5% CO_2_, 37°C in DMEM (Sigma Aldrich, St. Louis, Missouri, USA) with 10% fetal bovine serum (FBS) (Invitrogen, Paisley, UK), 2 mM L‐glutamine, 100 U/ml penicillin, and 100 U/ml streptomycin (Lonza, Basel, Switzerland). Adherent cells were harvested with trypsin (Biosera, Nuaillé, France) and counted with the Bürker chamber (Assistent, Sondheim vor der Rhön, Germany). In the experiments, a stock solution of 5 mM perifosine (Sigma‐Aldrich, St. Louis, Missouri, USA) diluted in 50% ethanol (VWR International, Monroeville, Pennsylvania, USA) and/or a stock solution of 1.25 mM ABT‐737 (MedChemExpress, South Brunswick, New Jersey, USA), also diluted in 50% ethanol, were used to treat the cells.

### 2.2 The pH of the culture media

Experiments were performed in four different culture media mimicking the tumour microenvironment (normal pH/normoxia, acidic pH/normoxia, normal pH/hypoxia, acidic pH/hypoxia), as recently described in detail.^10^ Briefly, for hypoxic cell culture (1% O_2_), the BioSpherix I‐Glove cell culture chamber (BioSpherix, New York, USA) was used. Acidic conditions (pH 6.6) were simulated by pH modulation of RPMI 1640 media (Sigma‐Aldrich, St. Louis, Missouri, USA) with 20 mM lactic acid (LA). As a control, 20 mM sodium lactate (NaL) was added to the media with a pH of 7.4.

### 2.3 Three‐dimensional cell culture models

The formation of spheroids from the cell lines HT‐29 and HCT‐116 has been described previously.^10^, ^32^

### 2.4 Assays for cell proliferation and viability

#### 2.4.1 MTT assay

Metabolic activity of cells was measured using the MTT assay as recently reported.^10^ Cells (10^5^ cells/ml) were seeded in control and acidic media in normoxia or hypoxia for 72 h then, the depleted media were replaced with fresh media and the cells were treated with perifosine in combination with ABT‐737 (final concentrations of both: 2.5, 5, 10, 20 and 40 μM) for 48 h. Control cells were treated with 50% ethanol as solvent. The 3D models were treated with perifosine and/or ABT‐737 (final concentrations: 5–20 μM) for 48 h under normal conditions, as acidosis and hypoxia naturally occur in the 3D tumour models.^10^, ^33^ MTT assay was performed for both monolayers and spheroids as described.^10^

#### 2.4.2 ATP assay

Cells in 2D were adapted to culture media with different pH and oxygen values for 72 h and then treated with 5 μM perifosine, 5 μM ABT‐737 and their combination for 24 h. For spheroid induction, chemical doses were raised to a concentration of 20 μM and the exposure time was increased to 48 h. Quantification of cellular ATP was performed using the ATP assay kit (Cayman Chemicals, Ann Arbor, Michigan, USA) as previously described.^10^, ^34^

#### 2.4.3 Live/dead cells staining of spheroids

Spheroids were treated with a 20 μM final concentration of perifosine and/or 20 μM ABT‐737 for 48 h under normal conditions. Cell viability up to 100 μm from the spheroid surface was determined using calcein‐AM/propidium iodide live/dead cell staining and emitted fluorescence was detected using laser scanning confocal microscopy (LSCM) (TCS SP8, Leica Microsystems, Wetzlar, Germany) as described.^10^ The final ratio of dead to live cells was examined using TissueQuest software (TissueGnostics, Vienna, Austria).

### 2.5 Measurement of mitochondrial membrane potential

HT‐29 cells in monolayers were pretreated with NaL, LA containing medium in normoxia and hypoxia for 72 h, followed by media exchange and induction with perifosine (5 μM), ABT‐737 (5 μM) and their combination for 48 h. Then 2 μM final concentration of JC‐1 probe (Invitrogen, Paisley, UK) was added for 30 min and fluorescence of the probe was measured by flow cytometry at λex = 488 nm using FITC and PE filters.

### 2.6 Determination of ROS production

HT‐29 cells in 2D were pretreated in NaL‐ and LA‐containing media in normoxia and hypoxia for 72 hours. Then, the medium was replaced with fresh medium and the cells were induced by perifosine (5 μM), ABT‐737 (5 μM) alone or their combination for 48 hours. The reactive oxygen species (ROS) were determined with 2′,7′‐dichlorodihydrofluorescein diacetate (DCFH‐DA) (final concentration 1 μM, Sigma Aldrich, St. Louis, Missouri, USA) for 30 min. Cells to be cultured in hypoxia were exposed to this microenvironment during the addition of probe and cell harvest. Flow cytometry with λex = 488 nm and FITC filter was then used for ROS quantification.

### 2.7 Gel electrophoresis and immunoblotting

Cells (2 × 10^5^ cells/ml) were seeded in different culture media for 72 h. Then, the depleted media were replaced with fresh media and cells were treated with 5 μM perifosine and/or 5 μM ABT‐737 for 24 h or left untreated. For spheroids, 20 μM concentrations of both compounds were used (48 h). Cells were collected, lysed, and processed for gel electrophoresis followed by immunoblotting as described in Ref.[10, 35] Blots were probed with antibodies specific for Akt phosphorylated at Ser473 (p‐Akt), cleaved PARP (cv. PARP), phosphorylated histone 2A.X (p‐H2A.X) (CST4060, CST32563, CST80312S, Cell Signaling Technology, Beverly, Massachusetts, USA) and α‐tubulin (ab7291, Abcam, Cambridge, UK). To compare protein abundances between samples, densitometric analysis was performed using ImageJ software (U. S. National Institutes of Health, Bethesda, Maryland, USA).

### 2.8 Immunohistochemistry of spheroid sections

Spheroids were treated with perifosine and/or ABT‐737 (both 20 μM) for 48 h. Spheroids were then collected in cryomolds containing OCT (Tissue‐Tek® O.C.T.™ Compound, Sakura Finetek USA, St. Torrance, California, USA) and frozen at −80°C. Tissue sections were processed for immunohistochemical (IHC) analysis as recently described.^10^, ^32^ Sections were incubated with a mixture of primary antibodies, Ki‐67 (ab16667, Abcam, Cambridge, UK) and cv. PARP (CST 32563 S, Cell Signalling Technology, Beverly, Massachusetts, USA). Final images were acquired with LSCM and analysed using “peeling analysis software” that we developed previously.^32^, ^36^

### 2.9 Statistical data analysis

The distributions of the collected data were described in bar graphs with the mean and standard deviations (SDs). Statistical comparison for the viability tests was performed using the t‐test. Combination indexes (CIs) showing efficacy between drug combinations were evaluated based on the method Chou‐Talalay^37^ using CompuSyn software (Biosoft, Cambridge, UK). Extreme values of the mean effect values (0.02 < Fa < 0.99) were excluded from the analysis according to the recommendations of author.^37^ Drug–drug interactions were based on the value of CI defined by Feng et al.^38^ (Table 1).

**TABLE 1:** Definition of drug interactions based on the value of CI.^38^

CI	Interaction
>1.3	Antagonism
1.1–1.3	Moderate antagonism
0.9–1.1	Additive effect
0.8–0.9	Slight synergism
0.6–0.8	Moderate synergism
0.4–0.6	Synergism
<0.4	Strong synergism

For the statistical analysis of the signals obtained after the “peeling analysis”, the protocol presented by Machálková et al.^32^ was followed. Differences between median values of a given antibody abundance in spheroids were tested using the Mann–Whitney test and p‐values were adjusted using the Bonferroni correction for comparison of multiple tests. At least three independent experiments were performed.

## 3 RESULTS

### 3.1 The combination of perifosine and ABT‐737 effectively reduces the viability of colon cancer cells in monolayers

**FIGURE 1:**
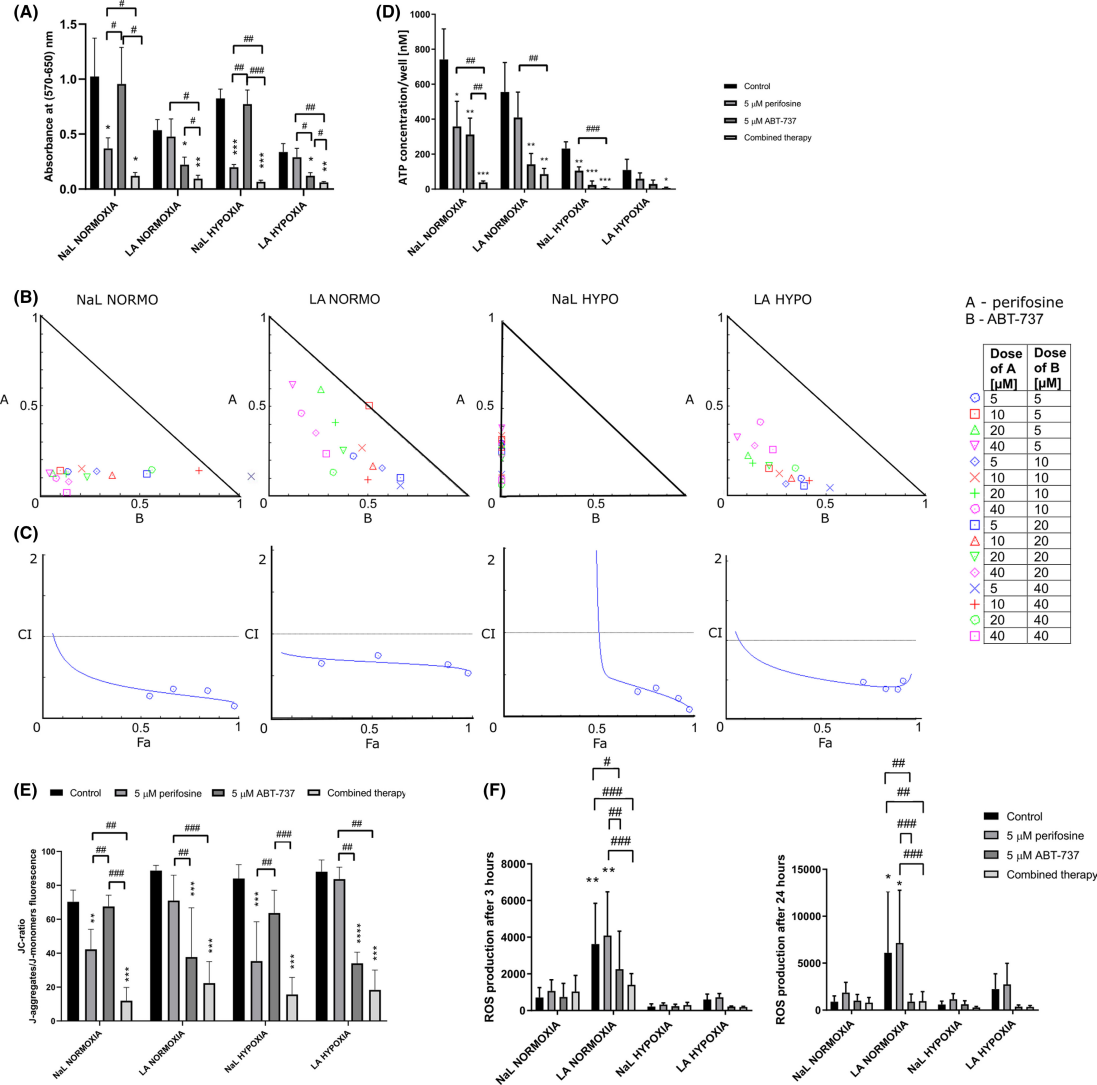
The effect of tumour environment on the cytotoxicity of perifosine and ABT‐737 to HT‐29 cells in monolayers. (A–C,E,F) Cells were pretreated in different tumour environments for 72 h and then treated with perifosine or ABT‐737 for 48 h. (A) Cytotoxicity was assessed by MTT. Results are presented as mean ± SD. (B) The isobolograms of perifosine and ABT‐737 in constant and non‐constant drug ratios in all environments tested. The diagonal line indicates additivity. Data points below the additivity line indicate synergy, data points above indicate antagonism. (C) Fa–CI plot of perifosine and ABT‐737 in constant drug ratio (1:1) in all tested tumour microenvironments. CI was plotted on the y‐axis as a function of efficacy (Fa) on the x‐axis. Drug dose combinations are shown in the table. (D) Cells were pretreated in different tumour environments for 72 h, then treated with perifosine/ABT‐737 for 24 h, and cytotoxicity was determined by ATP assay. Results are presented as mean of ATP level per well ± SD. (E) Mitochondrial membrane potential was measured as JC‐1 probe fluorescence using flow cytometry. (F) ROS level was assessed using DCFH‐DA staining and flow cytometry. Data are presented as mean ± SD. All the experiments were repeated at least three times. Significant differences between treatments were determined by *t*‐test; *p < 0.05, **p < 0.01, ***p < 0.001 for controls and induced cells (A,E,D) or for the similarly treated samples in NaL NORMOXIA and LA NORMOXIA (F) and #*p* < 0.05, ##*p* < 0.01, ###*p* < 0.001 for single drug and combination‐induced cells, unless otherwise indicated.

The environment of a tumour can critically influence the efficacy of chemotherapy. Therefore, the individual cytotoxicity of perifosine and ABT‐737 was tested for the colon cancer cell line HT‐29 in different tumour environments; pH 7.4, normoxia (NaL NORMO), pH 6.6, normoxia (LA NORMO), pH 7.4, hypoxia (NaL HYPO) and pH 6.6, hypoxia (LA HYPO). Cells were pretreated for 72 h at normal pH or in acidosis, in both normoxia and hypoxia, and induced for 48 h by perifosine (5 μM), ABT‐737 (5 μM) or by their combination and evaluated by MTT. Acidosis or acidosis combined with hypoxia decreased the cytotoxicity of perifosine to HT‐29 cells. In contrast, lower pH increased the efficacy of ABT‐737 (Figure 1A). The drug combination was effective to HT‐29 cells regardless of the microenvironment (Figure 1A).

Thus, our next objective was to investigate the interaction between perifosine and ABT‐737 in a tumour‐specific microenvironments. Cells were exposed to NaL NORMO, LA NORMO, NaL HYPO, LA HYPO for 72 h. Then, perifosine and ABT‐737 were added in non‐constant and constant drug ratios. After 48 h, cell viability was determined by MTT assay and Chou‐Talalay method was used to determine CI (Table 2).

**TABLE 2:** Combination indexes (CI) and fraction affected (Fa) values calculated for drug interactions in cells cultured in monolayers and exposed to different tumour‐specific environments and in 3D tumour settings

Dose of perifosine [μM]	Dose of ABT‐737 [μM]	NaL NORMO		LA NORMO		NaL HYPO		LA HYPO		3D	
		Fa	CI	Fa	CI	Fa	CI	Fa	CI	Fa	CI
5	5	0.548	0.271	0.249	0.651	0.709	0.304	0.727	0.473	0.277	0.827
10	5	0.683	0.240	0.191	1.014	0.813	0.326	0.795	0.366	0.436	0.641
20	5	0.818	0.190	0.474	0.857	0.900	0.297	0.859	0.336	0.642	0.595
40	5	0.894	0.170	0.822	0.741	0.930	0.390	0.910	0.380	‐	‐
5	10	0.539	0.421	0.433	0.730	0.715	0.296	0.831	0.363	0.395	0.573
10	10	0.669	0.357	0.533	0.739	0.803	0.347	0.843	0.386	0.543	0.478
20	10	0.822	0.249	0.693	0.749	0.902	0.289	0.895	0.314	0.687	0.520
40	10	0.914	0.177	0.906	0.628	0.944	0.298	0.878	0.578	‐	‐
5	20	0.564	0.659	0.699	0.766	0.741	0.256	0.867	0.444	0.502	0.455
10	20	0.720	0.476	0.788	0.697	0.832	0.282	0.880	0.426	0.710	0.271
20	20	0.843	0.338	0.882	0.632	0.922	0.219	0.907	0.379	0.825	0.288
40	20	0.928	0.220	0.950	0.592	0.955	0.234	0.928	0.422	‐	‐
5	40	0.571	1.176	0.905	0.722	0.847	0.125	0.895	0.566	‐	‐
10	40	0.684	0.938	0.945	0.591	0.916	0.121	0.908	0.500	‐	‐
20	40	0.798	0.707	0.976	0.458	0.974	0.063	0.918	0.500	‐	‐
40	40	0.980	0.149	0.981	0.529	0.980	0.093	0.936	0.490	‐	‐

In nearly all tested environments, synergistic interactions between both drugs were found (Figure 1B). In NaL NORMO, only a combination of 5 μM perifosine with 40 μM ABT‐737 led to moderate antagonism. In LA NORMO, the combination of 10 μM perifosine and 5 μM ABT‐737 revealed additivity. In NaL HYPO and LA HYPO, synergism was detected in a broad range of drug ratios (Figure 1B). Then the constant drug combinations (perifosine:ABT‐737 = 1:1) from Figure 1B were selected for computerized simulation of Fa–CI effect (Figure 1C). The Fa‐CI plots revealed synergism of this drug combination in broad range of Fa values in NaL NORMO, LA NORMO, and LA HYPO. Only in NaL HYPO, the simulation at low Fa showed antagonism. The highest cytotoxicity of the combination compared to uninduced controls was confirmed in NaL NORMO and LA HYPO by ATP assay (Figure 1D). In LA NORMO and NaL HYPO, the differences in ATP‐level in ABT‐737 and the combination‐treated samples were not significant. However, the trend towards the highest depletion of ATP levels in the combination‐exposed cells was evident (Figure 1D).

For the HCT‐116 cell line, synergism between perifosine and ABT‐737 was shown particularly in LA NORMO and NaL HYPO. In NaL NORMO and LA HYPO, drug interactions were mostly synergistic or additive (File S1A). In constant drug combinations, synergism was detected in all tested environments especially at higher Fa values (Fa ≥ 0.5) (File S1B). All combination indexes are shown in Table S1.

Then, we tested the effect of perifosine, ABT‐737, and their combination on mitochondrial potential and ROS production. Perifosine decreased mitochondrial potential at normal pH but was not effective at acidic pH. This is in contrast with ABT‐737, which caused changes in mitochondrial potential mainly at acidic pH. The combination of perifosine and ABT‐737 decreased mitochondrial potential in all tumour microenvironments (Figure 1E). Increased production of ROS was detected in uninduced controls and in perifosine‐treated samples at LA NORMO (Figure 1F). In other microenvironments, treatments had no effect on the level of ROS.

### 3.2 ABT‐737 alone and the combined treatment of perifosine and ABT‐737 induces apoptosis in monolayers formed by colon cancer cells

**FIGURE 2:**
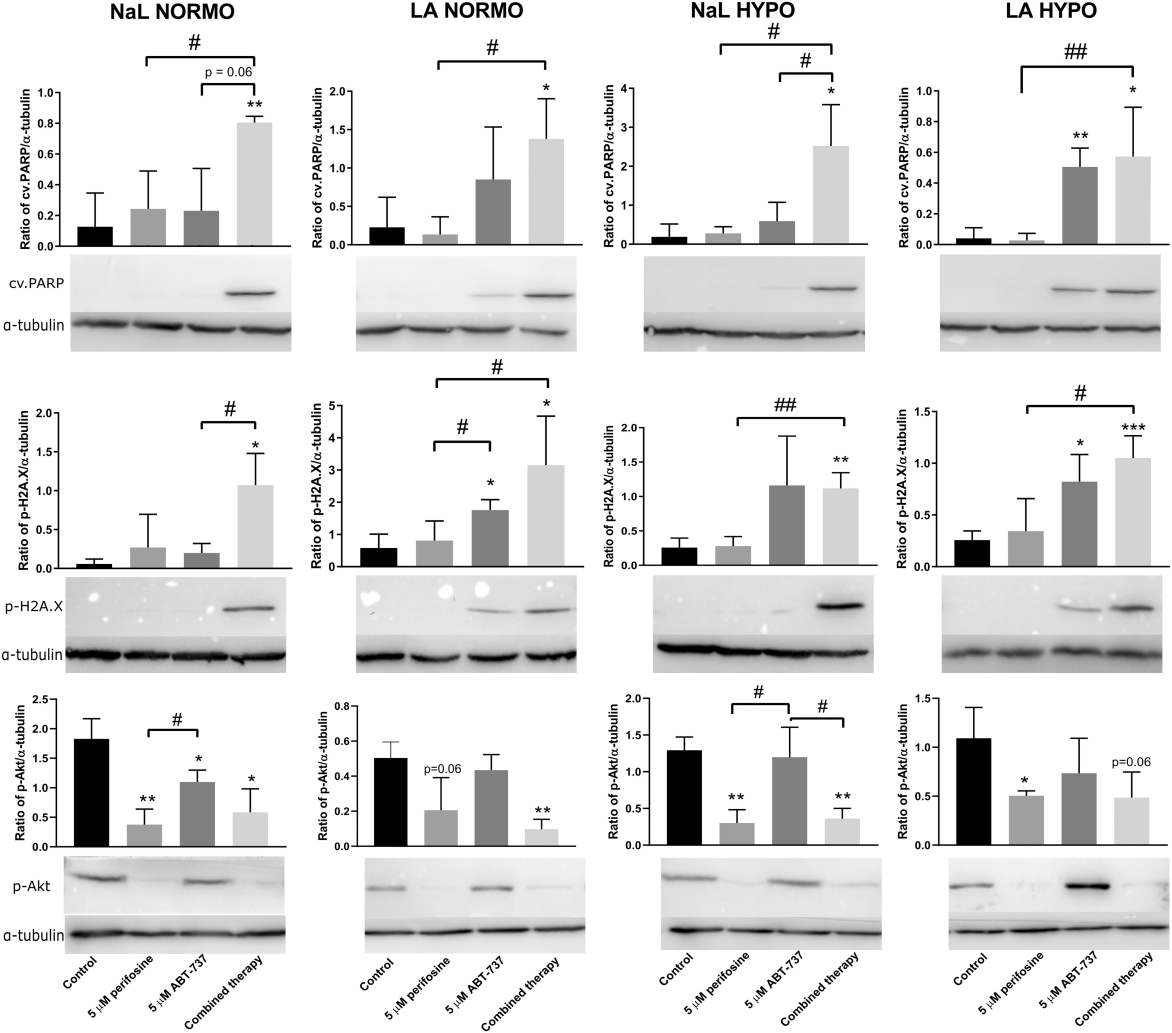
Immunoblotting of cv. PARP, p‐H2A.X, p‐Akt, and α‐tubulin intracellular levels. Cells in the monolayer were cultured for 72 h in different tumour environments and treated with perifosine, ABT‐737 or their combination for 24 h. Relative protein expression was assessed by densitometric analysis. Data are presented as mean ± SD. Significant differences were determined by *t*‐test; *p < 0.05, **p < 0.01, ***p < 0.001 for controls and induced cells and #*p* < 0.05, ##*p* < 0.01 for single agent cells and those induced by the combination. At least three independent experiments were performed.

First, the pro‐apoptotic effects of perifosine, ABT‐737 and their combination against HT‐29 monolayers were tested by analysis of cv.PARP level. ABT‐737 alone increased the level of cv. PARP in LA HYPO (Figure 2). A trend of increasing cv PARP in ABT‐737‐exposed cells was also observed in LA NORMO (Figure 2). In contrast, the combination increased cv. PARP regardless of the microenvironment to which the cells were exposed (Figure 2). P‐H2A.X is a marker of DNA damage and early apoptosis^39^; therefore, we examined its levels after perifosine and ABT‐737 exposure. ABT‐737 alone increased the levels of p‐H2A.X in LA NORMO and LA HYPO (Figure 2). In combination, the increase in the level of this protein was detected in all microenvironments (Figure 2). Perifosine alone or in combination effectively downregulated the phosphorylation of Akt kinase at Ser473 regardless of the environment, confirming its inhibitory effect on Akt kinase activity (Figure 2).

### 3.3 Perifosine and ABT‐737 synergistically decrease viability of colon cancer cells in 3D tumour models

**FIGURE 3:**
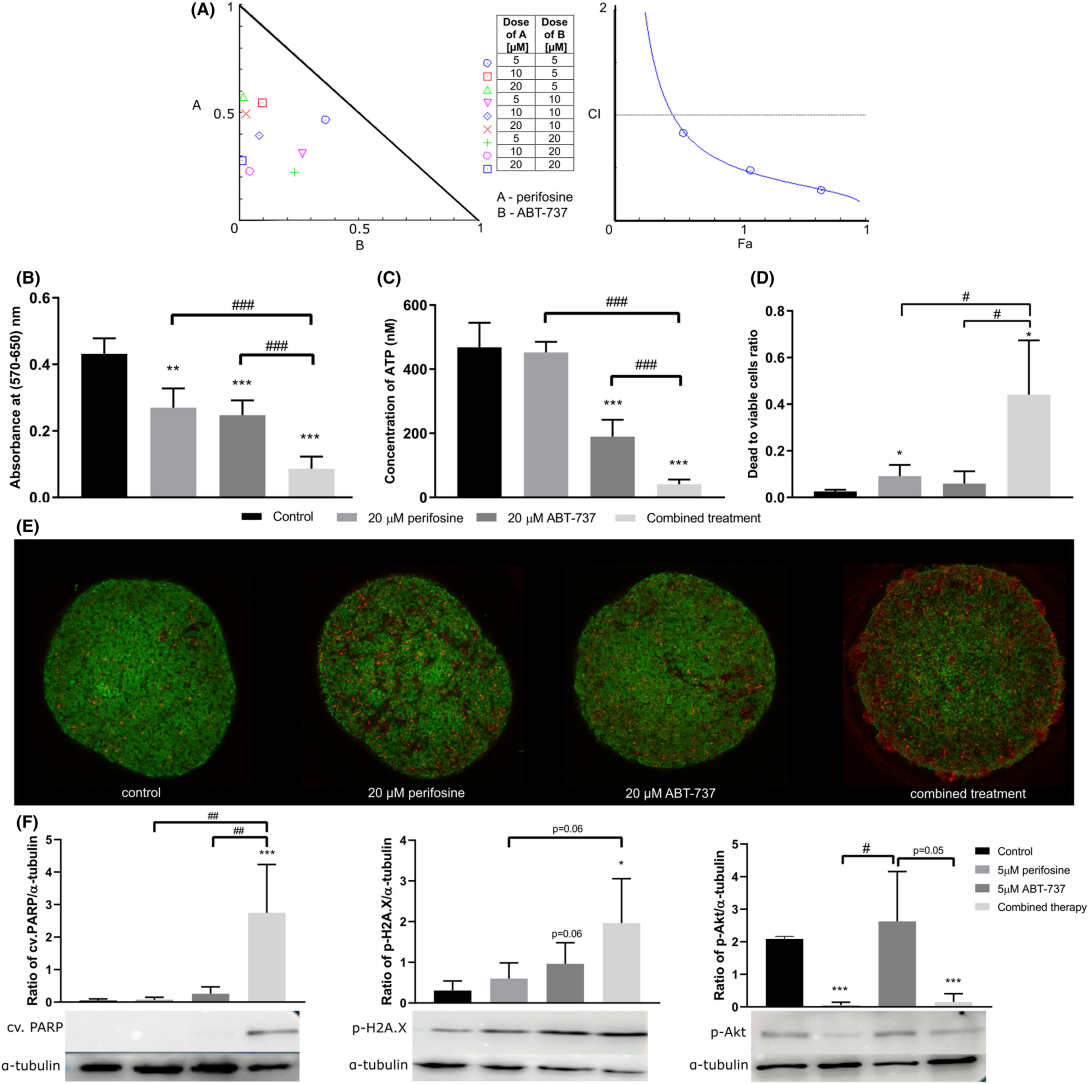
Cytotoxicity of perifosine and ABT‐737 in 3D tumour models derived from HT‐29 cells. (A–E) Spheroids were treated with perifosine, ABT‐737 and their combination for 48 h. (A) Cell viability was assessed using the MTT assay to perform isobologram analyses of perifosine and ABT‐737 in constant and non‐constant drug ratios. The diagonal line indicates additivity. Data points below the additivity line indicate synergy, data points above indicate antagonism (left). Fa–CI plot for a constant drug ratio of perifosine and ABT‐737 (1:1). CI was plotted on the y‐axis as a function of efficacy (Fa) on the x‐axis (right). Drug dose combinations are shown in the table. (B,C) Cytotoxicity of perifosine and ABT‐737 assessed by MTT and ATP assays. (D) Cytotoxicity of perifosine and ABT‐737 calculated as the ratio of dead and live cells after calcein‐AM/propidium iodide staining. (E) Images of spheroids induced by perifosine, ABT‐737 and their combination obtained by LSCM; live cells were stained with calcein‐AM (green) and dead cells with propidium iodide (red). (F) Spheroids were treated with perifosine, ABT‐737 or combination for 48 hours. After treatment, cells were lysed and proteins were subjected to immunoblotting with antibodies specific to cv. PARP, p‐H2A.X, p‐Akt, and α‐tubulin. Relative protein expression was assessed by densitometric analysis. Data are presented as mean ± SD. At least three independent experiments were performed. Significant differences were determined by *t*‐test; *p < 0.05, **p < 0.01, ***p < 0.001 for controls and induced spheroids and #*p* < 0.05, ##*p* < 0.01, ###*p* < 0.001 for single agent spheroids and those induced by the combination.

To further approximate real in vivo conditions, we tested drug combinations using 3D tumour models (spheroids). First, we obtained spheroids from the HT‐29 cell line and treated them with perifosine and ABT‐737 at concentrations of 5–20 μM. The viability of the cells was evaluated by MTT assay, and the combination indexes were counted for a non‐constant and a constant drug combination (Table 2). The results showed the synergistic effect of the combination therapy at all tested concentrations on the spheroids obtained from HT‐29 (Figure 3A). Again, the Fa–CI plots showed the synergism of the drug combination in a wide range of Fa values, while the simulation detected the antagonism only at low Fa (Figure 3A).

Next, we checked the cytotoxic potential of perifosine (20 μM), ABT‐737 (20 μM) and their combination on HT‐29 spheroids using MTT (Figure 3B), ATP (Figure 3C) and propidium iodide/calcein‐AM staining (Figure 3D,E). MTT and propidium iodide/calcein‐AM staining demonstrated the decrease in metabolic activity and viability after a single perifosine treatment (Figure 3B,D,E). ABT‐737 alone significantly decreased both metabolic activity and ATP levels (Figure 3B,C). The combined treatment with perifosine and ABT‐737 was most effective in down‐regulating ATP level, metabolic activity and inducing cell death compared to the single treatments (Figure 3B–E).

The highest efficacy of perifosine/ABT‐737 combination was demonstrated in spheroids derived from HCT‐116 cell line by MTT assay as well (File S2).

### 3.4 Drug combination induces apoptosis in spheroids

Next, we compared the levels of the above‐mentioned proteins in 3D spheroids by immunoblotting (Figure 3F). The combined treatment increased p‐H2A.X and especially cv. PARP levels. The level of phosphorylated Akt (Ser473) was downregulated by perifosine alone as well as by drug combination.

### 3.5 The combined therapy effectively increases cell apoptosis but stimulates proliferation of survivors

**FIGURE 4:**
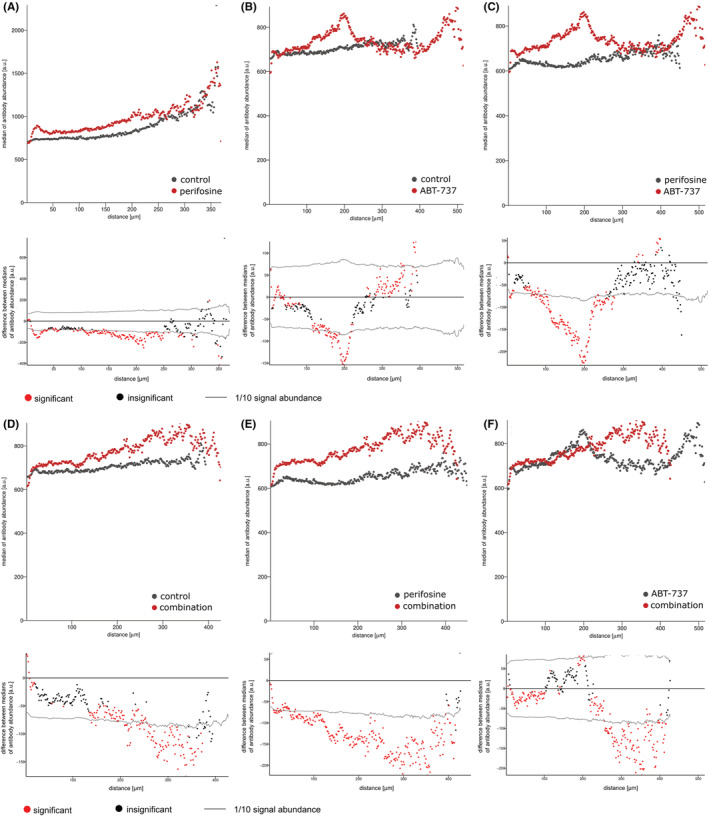
The “peeling analysis” of cv. PARP distribution in spheroids after perifosine, ABT‐737 and combined treatments. (A–F, top rows) Plots showing the distribution of cv. PARP (median per peel) in the different treated spheroid sections from edge to center. (A–F, bottom rows) Differences between the median values of cv. PARP abundance in the control and treated spheroids or the differentially induced spheroids. The median value of the 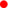
‐marked spheroid was subtracted from the 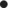
‐marked spheroid in each peel. The zero point on the X‐axis coincides with the outer edge of the spheroid. Significant (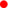) and insignificant (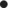) differences in medians are distinguished, as determined by the Mann–Whitney test (*p* < 0.05). Results are representative of at least three independent experiments.

**FIGURE 5:**
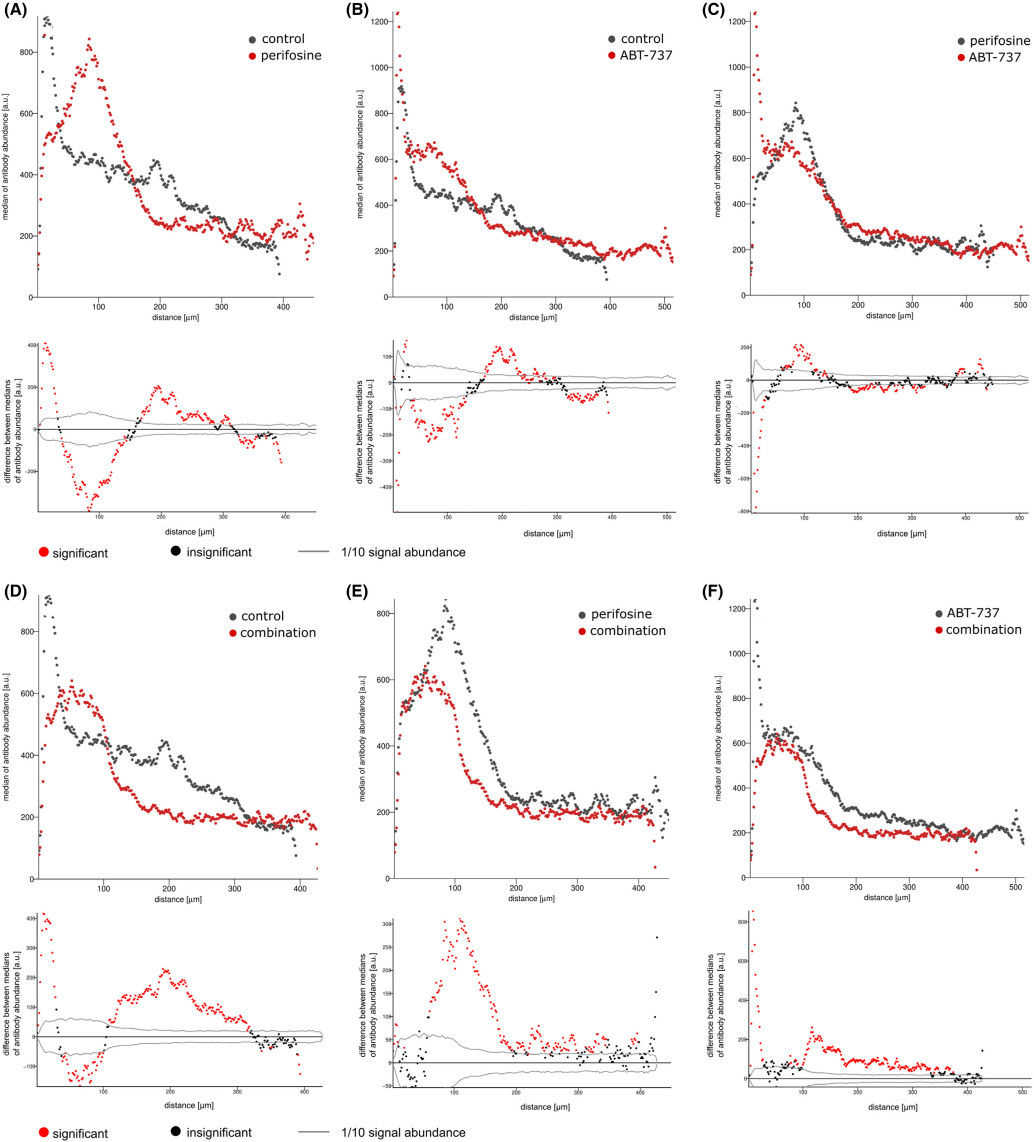
The “peeling analysis” of Ki‐67 distribution in spheroids after perifosine, ABT‐737 and combined treatments. (A–F, top rows) Plots showing the distribution of Ki‐67 (median per peel) in the different treated spheroid sections from edge to center. (A–F, bottom rows) Differences between median values of Ki‐67 abundance in control and treated spheroids or differentially induced spheroids. The median value of 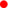
‐marked spheroid was subtracted from the 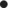
‐marked spheroid in each peel. The zero point on the X‐axis coincides with the outer edge of the spheroid. Significant (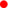) and insignificant (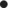) differences in medians are distinguished, as determined by the Mann–Whitney test (*p* < 0.05). Results are representative of at least three independent experiments.

Although knowledge of specific proteins in the cell pool is important, their distribution in the 3D tumour model is more relevant in the tissue context. We therefore followed changes in the distribution of the proliferation marker Ki‐67 and the apoptosis marker cv. PARP after treatment with perifosine and/or ABT‐737. We embedded the treated spheroids and control spheroids in OCT media, made cryosections, and stained the proteins of interest with IHC. Images of the spheroids showing the distribution of Ki‐67 and cv. PARP after the different treatments were taken with LSCM and are shown in File S3. The distribution of markers was assessed by “peeling analysis” from the edge of the spheroid to its center (Figures 4, 5; top rows), and the statistically significant changes are shown in Figures 4, 5; bottom rows. After 48 h, treatment with perifosine alone affected the level of cv. PARP first in the peripheral region up to 50 μm from the boundary and then slowly towards the central parts, compared to the control spheroid (Figure 4A). Treatment with ABT‐737 alone appears to be effective mainly in the 120–220 μm region (Figure 4B). Finally, we found that the combination of perifosine with ABT‐737 is most effective for upregulating cv. PARP. We detect increased levels of cv. PARP along the entire spheroid segment compared towith uninduced controls and perifosine‐induced spheroids. Compared with ABT‐737‐exposed spheroids, the combination was effective for increasing cv. PARP in the range 20–100 μm from the spheroid boundary and to an even greater extent deeper than 200 μm (Figure 4D–F). In all cases, we observed a slow increase of cv. PARP in the central part of the spheroids compared to the spheroid boundary. This is likely due to the presence of naturally apoptotic/necrotic cells in this region (Figure 4A–F).

For the Ki‐67 marker, we found a local decrease in proliferating cells in the marginal area at 48 hours after treatment with perifosine alone or in combination compared to controls and ABT‐737 (Figure 5A,C,D,F). Interestingly, there was a shift of the proliferating zone from the edge of the spheroid to the range of 50 to 100–180 μm after treatment with perifosine and the combined treatment compared to the uninduced controls (Figure 5A,D). Similarly, we also observed the shift in proliferating cells after treatment with ABT‐737 but there was also an increase in Ki‐67 positive cells in the edge region (Figure 5B). In all samples, there are only small amounts of proliferating cells in the central part of the spheroid (300 μm and deeper) (Figure 5A–F).

## 4 DISCUSSION

Drug resistance is a common problem in cancer therapies. Exposure of cancer cells to an acidic environment and overexpression of anti‐apoptotic proteins are some of the mechanisms that lead to drug resistance.^12^, ^40^ It is known that the acidic environment of tumours and hypoxia decreases the toxicity of drugs, including perifosine, to colon cancer cell lines.^9^, ^10^, ^41^ On the other hand, other compounds, such as ABT‐737, are more effective under acidic and hypoxic conditions.^14^, ^23^ We confirmed the cytotoxicity of ABT‐737 for colon carcinoma cell line HT‐29 in acidosis and acidosis associated with hypoxia.

In order to find effective treatment strategies, different drug combinations are being explored, with an advantage to those that can act independently of unfavourable tumour‐specific conditions. In this study, we mainly observed synergistic toxicity of combined therapy with perifosine and ABT‐737 in colon cancer cell lines. Previously, their reciprocal combination had shown synergistic activation of apoptosis in lung cancer cells.^42^ However, there is no evidence of toxicity of this combination therapy to CRC cells cultured in both monolayers and spheroids and in different tumour environments.

However, we have discovered several exceptions to the general synergistic mode of interaction, demonstrating that drug–drug interactions can be dose‐, cell line‐, tumour microenvironment and Fa‐dependent. First, the combination of perifosine and ABT‐737 can have synergistic, additive, or even antagonistic effects, depending on the drug dose and ratio. This fact may be explained by dose‐ and ratio‐dependent inhibition of cell proliferation or cell signalling pathways. Importantly, drug combinations in which one of the drugs is in excess concentration also appear to be less efficient, possibly due to the high toxicity of the single treatment. Second, the same drug dose may act synergistically in one microenvironment and antagonistically in cells cultured under different conditions (e.g., 5 μM perifosine/40 μM ABT‐737, HT‐29 cells in NaL NORMO and LA NORMO), possibly due to changes in the activity of targeted proteins that depend on the tumour microenvironment.^14^, ^43^ Third, the nature of the drug interaction could be cell line specific (e.g., HT‐29 and HCT‐116 cells in LA HYPO), with the effect likely due to the different mutational profiles of these cell lines. Fourth, simulations for constant drug ratios revealed that interaction between perifosine and ABT‐737 can be antagonistic, but the antagonism was detected especially at low Fa values (e.g., HT‐29 NaL HYPO). In higher Fa, the synergy between perifosine and ABT‐737 was determined. Since synergy at high Fa values is more relevant to cancer therapy than at low values,^37^ these results do not contradict the possible use of the combination in the treatment of cancer.

Our results showed that single treatment with ABT‐737 in acidosis and combined therapy of perifosine and ABT‐737 resulted in DNA damage, as evidenced by p‐H2A.X. The effect of ABT‐737 (alone or in combination with other drugs) on DNA damage has been reported previously.^44^, ^45^, ^46^ In addition, it has been found that treatment with ABT‐737 at sublethal concentrations can cause DNA damage that leads to gene instability and promotes carcinogenesis.^46^, ^47^ The Akt protein is involved in the DNA repair machinery,^48^ and therefore its inhibition could affect the viability of cells with damaged DNA. Regardless of the Akt kinase, perifosine promotes degradation of RAD51, key mediator of homologous recombination mediated DNA double‐strand break repair. Thus, perifosine can reduce DNA repair capability in cells exposed to DNA‐damaging agents ABT‐737.^49^, ^50^

In addition to the level of expressed proteins in the pooled cell samples, it is also necessary to determine their distribution in the tumour models. For this purpose, spheroids clearly represent a better model than cells in monolayer.^51^ The cells that form the tumour are often heterogeneous and respond differently to therapy. The uneven distribution of drugs in the tumour may contribute to the formation of resistant areas.^52^, ^53^, ^54^ Therefore, we investigated the protein expression of specific markers in spheroids using IHC. For the analysis of IHC markers, we used a recently developed semi‐automated “peeling analysis” software that allows the evaluation of signals from all cells from the edge to the center of the spheroid.^32^, ^36^ Using this tool, we determined the distribution of cv. PARP, a marker of intrinsic apoptosis, and Ki‐67, a proliferative marker, after treatment with perifosine and/or ABT‐737. Compared to blank spheroid, treatment with the combination was effective in inducing apoptosis in spheroids, with the highest amount of cv. PARP detected. Perifosine alone increased apoptotic markers first in the peripheral region and then slowly along the spheroid section. ABT‐737 alone increased cv. PARP mainly in the region 120–220 μm from the spheroid boundary. In all spheroids, there was an increase in cv. PARP levels towards the center, probably due to the inherently unfavourable environment.^51^ Analysis of the changes in proliferation after treatment provided new insights, as treatment with ABT‐737 led to a rapid increase in Ki‐67 levels at the spheroid boundary (up to 100 μm). We have already observed that the proliferation zone of the spheroid shifts a little from the edge to the deeper layers of the spheroid in response to drug penetration,^10^ but the increase in Ki‐67 positivity after treatment with ABT‐737 is quite remarkable. The combined treatment resulted in a further decrease in proliferation, but the shift of the proliferation zone towards the center of the spheroid is still visible. We hypothesize that both single and combined treatment with ABT‐737 help to loosen cell‐to‐cell adhesion, which could lead to better availability of nutrients from the medium and possible awakening of dormant/sleeping cells in the deeper layers. These results highlight the need to use efficient drug concentrations at proper time intervals to prevent chemotherapy failure.

## 5 CONCLUSION

In this study, we confirmed the crucial role of tumour‐specific environment in the cytotoxicity of perifosine and showed that perifosine and the BCL‐2 family inhibitor, ABT‐737, have a synergistic cytotoxic effect on colon cancer cell lines in any environment and dimensionality. Thus, the combined therapy of perifosine and ABT‐737 could be considered as a potential treatment strategy for colon cancer.

## AUTHOR CONTRIBUTIONS

**Barbora Adamová:** Data curation (equal); investigation (equal); methodology (equal); writing – original draft (equal). **Petr Benes:** Formal analysis (equal); funding acquisition (equal); project administration (equal); supervision (supporting); writing – original draft (equal). **Jan Smarda:** Formal analysis (equal); funding acquisition (equal); project administration (equal); supervision (supporting); writing – original draft (equal). **Jarmila Navrátilová:** Conceptualization (lead); data curation (equal); funding acquisition (equal); investigation (equal); methodology (equal); project administration (equal); supervision (lead); writing – original draft (equal). **Kamila Říhová:** Investigation (equal). **Jana Pokludová:** Investigation (equal).

## FUNDING INFORMATION

This work was supported by the European Regional Development Fund ‐ Project ENOCH (No. CZ.02.1.01/0.0/16_019/0000868) and and the project National Institute for Cancer Research (Programme EXCELES, ID Project No. LX22NPO5102) ‐ Funded by the European Union ‐ Next Generation EU.

## CONFLICT OF INTEREST

The authors declare that they have no conflict of interest.

## Supporting information


File S1.

File S2.

File S3.

Table S1.

## Data Availability

The raw data supporting the conclusions of this article will be made available by the authors upon reasonable request.
