# Supplementary material for: Synergistic cytotoxicity of perifosine and ABT‐737 to colon cancer cells
Source: J Cell Mol Med. 2022 Dec 15;27(1):76–88. doi: 10.1111/jcmm.17636 (PMC9806293; doi:10.1111/jcmm.17636)
Supplement: Supplementary file 1 — File S1. [file JCMM-27-76-s002.docx]

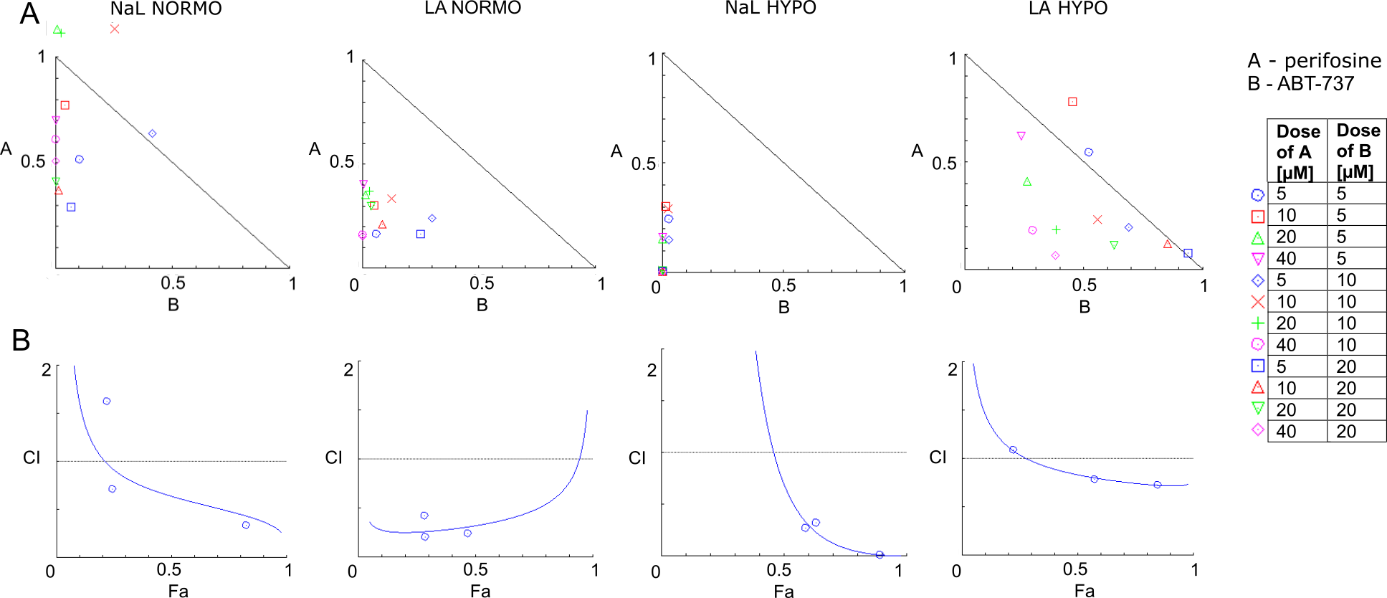


***Support file 1: The influence of tumor environment on the cytotoxicity of perifosine and ABT-737 on HCT-116 cells in monolayers***

*(A,B) Cells were pretreated in different tumor environments for 72 hours and then treated with different concentrations of perifosine/ABT-737 for 48 hours.* *Cytotoxicity was assessed by MTT.* *(A) The isobolograms of treatment with perifosine and ABT-737 in constant and non-constant drug ratios in all environments tested. (B) Fa-CI plot for constant drug ratio of perifosine and ABT-737 (1:1). CI was plotted on the y-axis as a function of efficacy (Fa) on the x-axis. Drug dose combinations are shown in Supporting Table 1.*
