# Supplementary material for: Synergistic cytotoxicity of perifosine and ABT‐737 to colon cancer cells
Source: J Cell Mol Med. 2022 Dec 15;27(1):76–88. doi: 10.1111/jcmm.17636 (PMC9806293; doi:10.1111/jcmm.17636)
Supplement: Supplementary file 2 — File S2. [file JCMM-27-76-s001.docx]

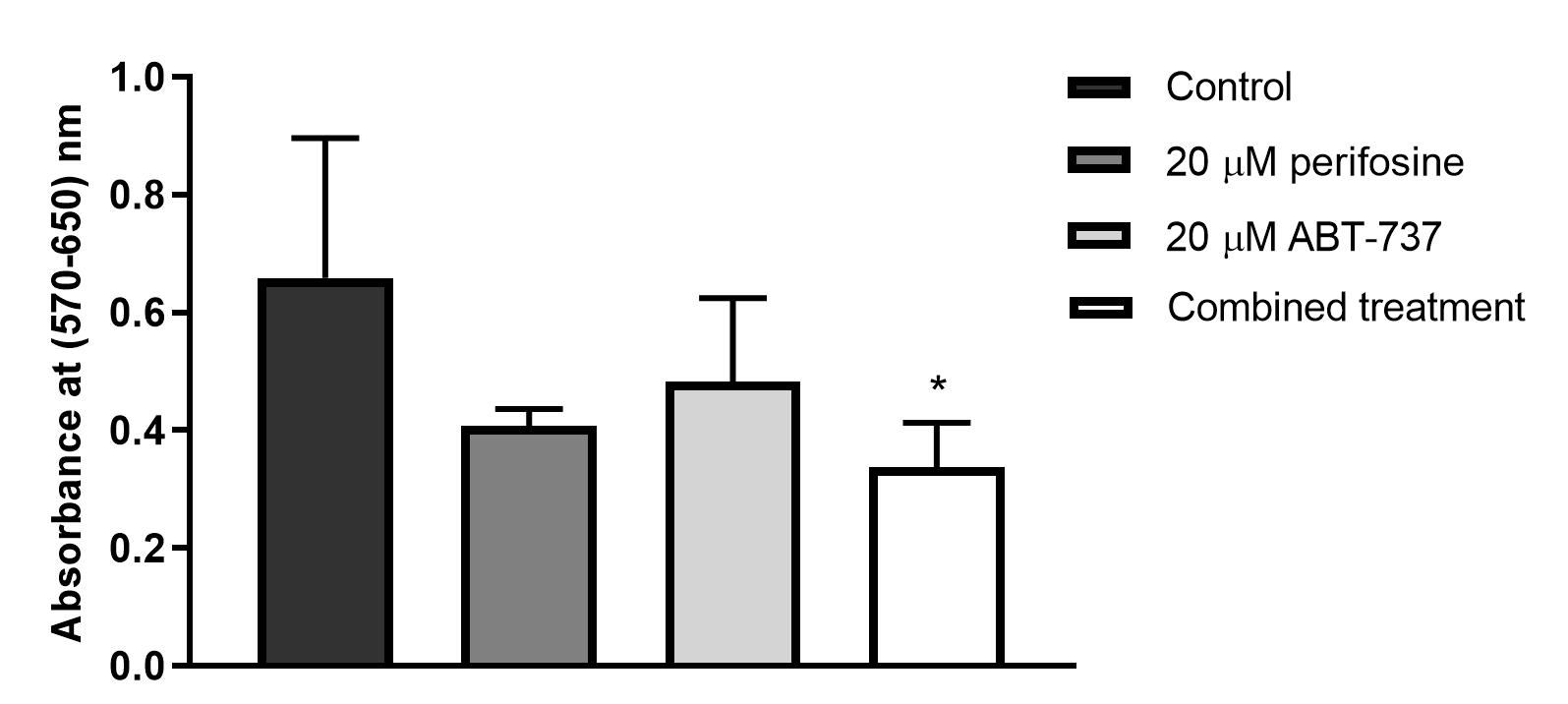


***Support file 2: The cytotoxicity of perifosine, ABT -737 and their combination in 3D tumor models derived from the HCT-116 cell line was evaluated after 48 hours of treatment using MTT.***

*Data are presented as mean +/- SD. Significant differences between control and treated spheroids were determined by t-test; *p <0.05, **p <0.01, ***p <0.001.*
