# Supplementary material for: Synergistic cytotoxicity of perifosine and ABT‐737 to colon cancer cells
Source: J Cell Mol Med. 2022 Dec 15;27(1):76–88. doi: 10.1111/jcmm.17636 (PMC9806293; doi:10.1111/jcmm.17636)
Supplement: Supplementary file 3 — File S3. [file JCMM-27-76-s004.docx]

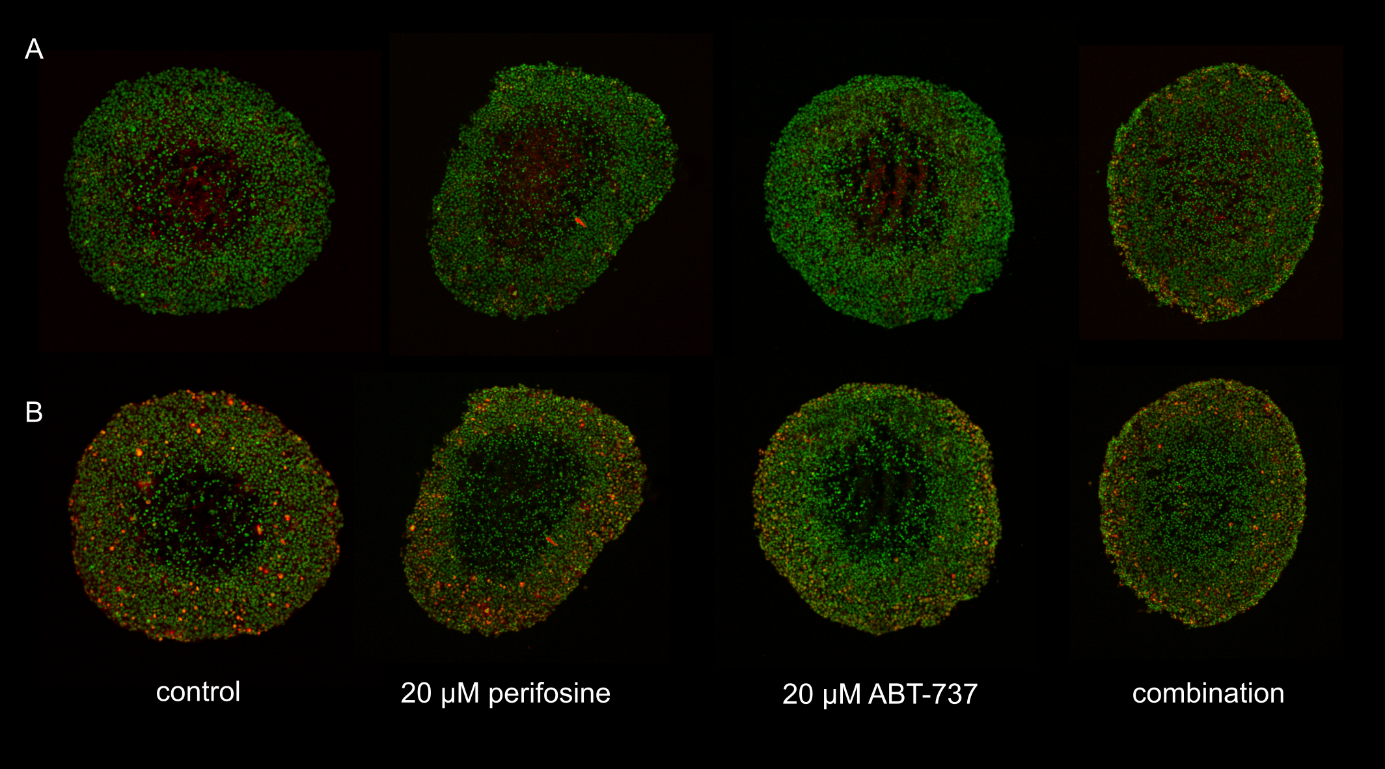


***Support file 3: IHC images of spheroids obtained by LSCM.***

*IHC images show the signal from (A) cleaved PARP/(B) Ki-67 (red) and nuclei (green) for control and perifosine/ABT-737-treated spheroids.*
