# Supplementary material for: Synergistic cytotoxicity of perifosine and ABT‐737 to colon cancer cells
Source: J Cell Mol Med. 2022 Dec 15;27(1):76–88. doi: 10.1111/jcmm.17636 (PMC9806293; doi:10.1111/jcmm.17636)
Supplement: Supplementary file 4 — Table S1. [file JCMM-27-76-s003.docx]

Support table 1: The combination indexes (CI) calculated for drug interactions in different tumor-specific environments.

| **Dose of perifosine [µM]** | **Dose of**  **ABT-737 [µM]** | **NaL NORMO** | | **LA NORMO** | | **NaL HYPO** | | **LA HYPO** | |
| --- | --- | --- | --- | --- | --- | --- | --- | --- | --- |
|  |  | **Fa** | **CI** | **Fa** | **CI** | **Fa** | **CI** | **Fa** | **CI** |
| 5 | 5 | 0.244 | 0.617 | 0.286 | 0.226 | 0.589 | 0.272 | 0.223 | 1.069 |
| 10 | 5 | 0.343 | 0.813 | 0.303 | 0.353 | 0.630 | 0.316 | 0.287 | 1.236 |
| 20 | 5 | 0.477 | 1.121 | 0.427 | 0.366 | 0.735 | 0.157 | 0.605 | 0.676 |
| 40 | 5 | 0.862 | 0.702 | 0.564 | 0.408 | 0.779 | 0.160 | 0.670 | 0.857 |
| 5 | 10 | 0.183 | 1.055 | 0.216 | 0.540 | 0.630 | 0.178 | 0.443 | 0.889 |
| 10 | 10 | 0.219 | 1.389 | 0.283 | 0.460 | 0.633 | 0.318 | 0.573 | 0.794 |
| 20 | 10 | 0.478 | 1.132 | 0.414 | 0.403 | 0.892 | 0.007 | 0.771 | 0.572 |
| 40 | 10 | 0.887 | 0.612 | 0.764 | 0.162 | 0.930 | 0.004 | 0.874 | 0.469 |
| 5 | 20 | 0.456 | 0.357 | 0.284 | 0.417 | 0.852 | 0.005 | 0.674 | 1.015 |
| 10 | 20 | 0.641 | 0.386 | 0.381 | 0.301 | 0.873 | 0.006 | 0.723 | 0.975 |
| 20 | 20 | 0.827 | 0.414 | 0.467 | 0.337 | 0.892 | 0.008 | 0.848 | 0.741 |
| 40 | 20 | 0.914 | 0.914 | 0.772 | 0.155 | 0.930 | 0.004 | 0.950 | 0.449 |
